# *Copitarsia decolora* Guenée (Lepidoptera: Noctuidae) females avoid larvae competition by detecting larvae damaged plants

**DOI:** 10.1038/s41598-020-62365-5

**Published:** 2020-03-27

**Authors:** Humberto Reyes-Prado, Alfredo Jiménez-Pérez, René Arzuffi, Norma Robledo

**Affiliations:** 1 Laboratorio de Ecología Química, EES Jicarero, Universidad Autónoma del Estado de Morelos, El Jicarero, Jojutla de Juárez, C.P, 62909 Morelos, México; 2 Laboratorio de Ecología Química de Insectos, Centro de Desarrollo de Productos Bióticos (CEPROBI), Instituto Politécnico Nacional, Km. 8.5 Carretera Yautepec-Jojutla de Juárez, Yautepec, Morelos C.P, 62731 México

**Keywords:** Chemical ecology, Plant signalling, Chemical ecology, Plant signalling, Plant signalling

## Abstract

Herbivory insects can discriminate the quality of a host plant for food or oviposition, by detecting the volatile organic compounds (VOC’s) released by the plant, however, damaged plants may release a different VOC’s profile modifying the insects’ response. We tested if the VOC’s profile from damaged plants affected the response of *Copitarsia decolora* as these moths oviposit preferably around undamaged host plants. We assessed the response in wind tunnel conditions of *C.* *decolora* mated females to volatiles collected by dynamic headspace from 30–40 d old cabbage undamaged plants and mechanical and larval damaged plants. Headspace volatile compounds from undamaged cabbage plants were more attractive to mated females than those from larval and mechanical damaged cabbage plants. Moths stimulated with headspace volatiles from undamaged plants performed more complete flight and ovipositor displays than those moths stimulated with headspace volatiles from damaged cabbage plants. A mixture of synthetic compounds identified from undamaged cabbages elicited similar antennal and wind tunnel responses in mated females as headspace volatiles from undamaged cabbage plants. *C. decolora* females may discriminate between damaged and undamaged host plants by detecting their VOC’s profiles as a strategy to avoid unsuitable plants for their offspring increasing their fitness.

## Introduction

Female moths recognize their host plants by specific ratios/concentrations of volatile organic compounds (VOCs) from a wide variety of plants^1,2^. Plants regulate their VOCs emission rate according to various factors during the day and night^3,4^. Emission of constitutive VOCs from undamaged plants provides clues to herbivory insects to select their host for oviposition^4^, however, when plants are damaged, whether by abiotic factors as wind or biotic factors as herbivory from insects, VOCs emission rates may vary qualitatively and quantitatively^1,5,6^. Herbivore insect host-seeking behavior is modulated by VOCs release either by plants damaged by conspecifics (most of the time larvae), other herbivores, mechanical damaged or the presence of eggs on the plant^7^.

It has been reported that mechanical damage does not induce a significant increase in the emission of VOCs in *Zea mays* L. and Lima bean *Phaseolus lunatus* Linnaeus^8–10^. Most studies on volatiles of host plants have focused on the VOCs emitted by plants damaged by herbivorous insects and their effect on conspecifics and the attraction of predators and parasitoids as an indirect defense mechanism^11,12^. In moths, females select the host plant for oviposition when it is suitable for the development of the larvae^13,14^. Females, therefore, may reduce the number of eggs placed on the host plant if it is not in optimum condition for the development of the offspring, thus avoiding larvae competition for food and the risk of parasitism or predation^15,16^. For example, *Spodoptera littoralis* Boisduval, females reduced oviposition on alfalfa *Medicago sativa* Linnaeus and cotton *Gossypium hirsutum* Linnaeus when these plants are herbivore-damaged^17^.

In the moth *Copitarsia decolora* Guenée, an important pest of cruciferous plants, particularly cabbage (*Brassica oleracea* var. capitata Linnaeus), which is widely distributed in America and quarantined for the United States^18^, females deposited significantly more eggs around undamaged cabbage plants than on the undamaged plant^19^, but there are no reports regarding the olfactory cues involved in the search for its host plant and the modification of this behavior due to the damage to the plant. This study reports the effect of headspace volatiles from damaged and undamaged cabbage plants on the attraction and electrophysiological responses of *C. decolora* females, and the chemical compounds attractive to mated females.

## Results

### Wind tunnel bioassays with headspace collection

**Figure 1:**
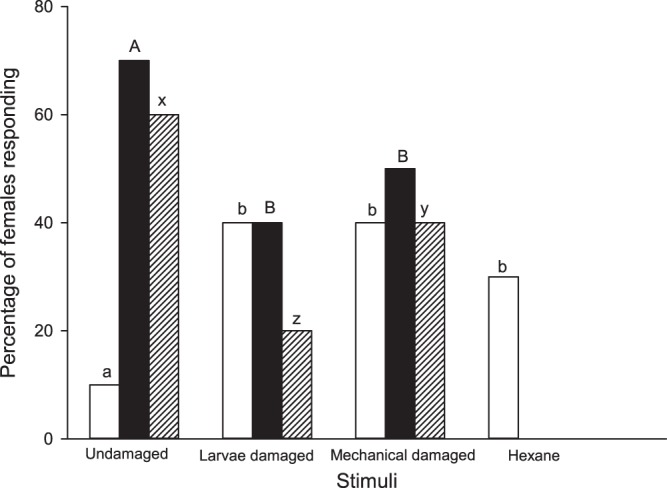
Percentage of mated females performing partial flight (empty bars), complete flight (solid bars) and ovipositor display (crossed bars) in the presence of headspace from undamaged cabbage and larvae- and mechanical-damaged cabbage plants in the wind tunnel. Hexane served as a control. Same color bars topped by the same letter are not significantly different (χ^2^, n = 10, P > 0.05).

Significantly more females performed more complete flight towards the headspace from undamaged plants than to the headspace from larvae-damaged plants (χ^2^ = 16,990; df = 1; P = 0.001) and to the headspace from mechanical-damaged plants (χ^2^ = 7,521; df = 1; P = 0.006). Similar number of females had a complete flight when exposed to the headspace from larvae and mechanical-damaged plants (χ^2^ = 1,636; df = 1; P = 0.201) (Fig. 1). No moths landed on the stimulus.

Similarly, significantly more females displayed their ovipositors in the presence of the headspace from undamaged plants than in the presence of the headspace from larvae-damaged plants (χ^2^ = 31,687; df = 1; P = 0.001) or in the presence of the headspace from mechanical-damage plants (χ^2^ = 7,220; df = 1; P = 0.007). However, more females displayed their ovipositors in the presence of the headspace from mechanical-damaged plants than to the headspace from larvae-damaged plants (χ^2^ = 8,595, df = 1; P = 0.003) (Fig. 1).

Females showed a similar number of partial flights in the presence of the headspace from larvae- and mechanical-damaged plants as to the hexane controls (χ^2^ = 1,780; df = 1; P = 0.182). Nevertheless, fewer females moths showed partial flights in the presence of the headspace from undamaged plants than from the headspace from larvae-damaged plants (χ^2^ = 22,427; df = 1; P = 0.001) and the headspace from mechanical-damaged plants (χ^2^ = 22,427; df = 1; P = 0.001) and hexane controls (χ^2^ = 11,281; df = 1; P = 0.001). None of the females evaluated to hexane presented a complete flight or ovipositor display (Fig. 1).

### Chemical identification

Fourteen compounds were identified from the cabbage headspace; the majority of compounds were present in the three headspaces collections. Qualitative differences were found and compounds such as Allyl isothiocyanate, α-pinene, Benzyl isothiocyanate, Geranyl acetone, and Octadecane were only identified in the headspace from damaged cabbage plants (Table 1).

**Table 1:** Chemical compounds identified by GC-MS in the headspace from undamaged, larvae-damaged and mechanical-damaged cabbage plants, in the wind tunnel.

Chemical compound	RT	CAS	MF	RI	HUC	HLD	HMD
Allyl isothiocyanate	4.13	57-06-7	C_4_H_5_NS	887 ^^a^		*	*
α-pinene	5.3	80-56-8	C_10_H_16_	982		*	*
6-methyl-5-hepten-2-one	7.56	110-93-0	C_8_H_14_O	974	*	*	
(Z) 3-hexenyl acetate	8.15	3681-71-8	C_8_H_14_O_2_	1004	*	*	*
Limonene	8.84	138-86-3	C_10_H_16_	1017	*	*	*
Nonanal	11.29	124-19-6	C_9_H_18_O	1057	*	*	*
Decanal	14.78	112-31-2	C_10_H_20_O	1203	*	*	*
Benzyl isothiocyanate	18.93	622-78-6	C_8_H_7_NS	1278		*	*
Tetradecane	21.19	629-59-4	C_14_H_30_	1400	*		*
Geranyl acetone	21.73	689-67-8	C_13_H_22_O	1458 ^^b^			*
Pentadecane	24.28	629-62-9	C_15_H_32_	1500	*		
Hexadecane	27.24	544-76-3	C_16_H_34_	1600	*	*	*
Heptadecane	30.02	629-78-7	C_17_H_36_	1700	*	*	*
Octadecane	31.44	593-45-3	C_18_H_38_	1800 ^^c^		*	*

RT = Retention Time, CAS = Chemical Abstracts Service, MF = Molecular Formula, RI = Kovats Retention Index, HUC = headspace from undamaged cabbage, HLD = headspace from larvae-damaged cabbage, HMD = headspace from mechanical-damaged cabbage. ^^a^Engel *et al*.^20^ ^^b^Leffingwell and Alford^21^. ^^c^Chung *et al*.^22^ *Identified in the headspace.

### Wind tunnel bioassays with headspace collection and synthetic compounds

**Figure 2:**
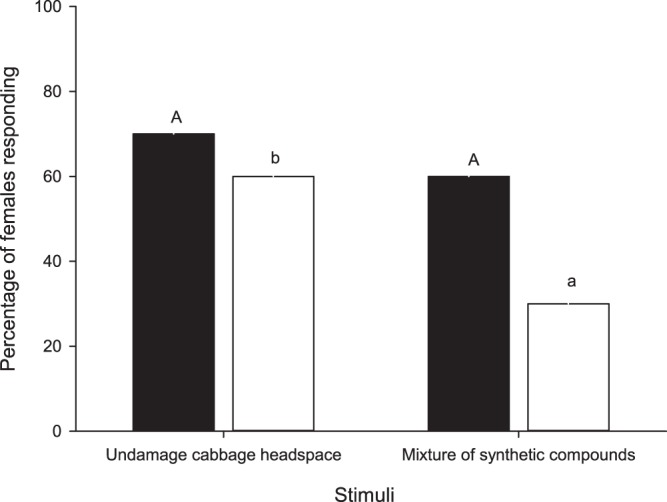
*Copitarsia decolora* mated females that performed a complete flight (solid bars) and ovipositor display (empty bars) responding to the headspace from undamaged cabbage plants and to the mixture of synthetic compounds in the wind tunnel. Same colored bars topped by the same letter are not significantly different (χ^2^, P > 0.05). n = 10.

Our artificial mixture corresponds well to the headspace collection from undamaged cabbage, since a similar number of females had a complete flight toward the headspace from undamaged cabbage compared to the mixture of synthetic compounds (χ^2^ = 1,780; df = 1; P = 0.182) but more females displayed their ovipositors in the presence of the headspace from undamaged cabbage than in the presence of the mixture of synthetic compounds (χ^2^ = 16,990; df = 1; P = 0.001) (Fig. 2). Moths did not show any orientation behavior or ovipositor display in the presence of hexane.

### Electroantennography

**Figure 3:**
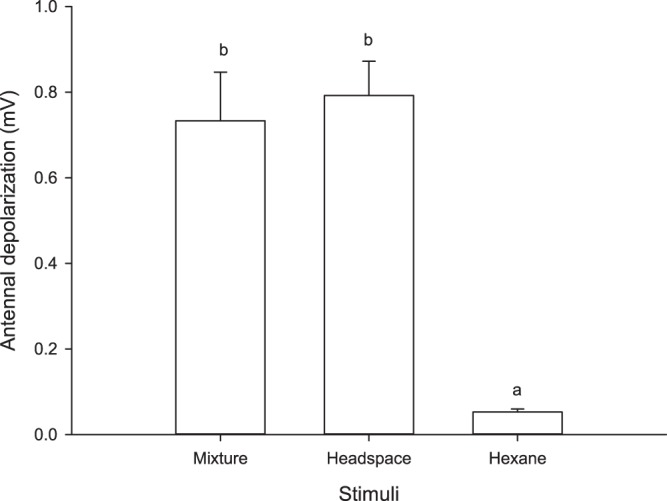
Mean antennal depolarization (± SEM) of mated females in response to the mixture of synthetic compounds, headspace from undamaged cabbage and hexane. Bars headed by the same letter are not significantly different, n = 12 (Tukey’s test, P > 0.05).

EAG responses to headspace from undamaged cabbage plants and the mixture of synthetic compounds were not significantly different between them, but these two stimuli elicited significantly different responses to the hexane (F = 28.413; df = 2, 35; P = 0.001) (Fig. 3).

## Discussion

Volatiles emitted by the host plant guide the moth to a suitable feeding and/or oviposition sites^23^. It is important to mention that our dynamic headspace volatile collection method reduced manipulation and successfully provided the volatiles produced by the live cabbage plant (damaged or undamaged). Our methodology is less intrusive and provides better results than that used by other authors. For example, Craveiro *et al*.^24^ used a microwave-assisted extraction method, that modified the original blends released by the plant. Rojas^25^ and Reddy and Guerrero^26^ obtained volatiles from human-cut cabbage leaves only. A qualitative difference was observed in the chemical profiles obtained from damaged and undamaged cabbage plants, so it is plausible to think that *C. decolora* females select its host by detecting some key compounds present on the VOCs of undamaged and damaged plants.

*Copitarsia decolora* females discriminate between headspace from damaged and undamaged plants by orienting towards the headspace from the undamaged plant and displaying their ovipositor. This behavior can be a sign of oviposition and could correspond to the preference of the female to lay eggs around undamaged plants than directly on them as it has been reported for this moth^19^.

The antennal and wind tunnel responses elicited by the headspace from undamaged cabbage were similar to the responses to the synthetic compound mixture, so this synthetic mixture corresponds well to the natural headspace. Compounds identified from the headspace from undamaged cabbage plants included (Z) 3-hexenyl acetate, which is a “green leaf volatile” found in the green leaves of cabbages^27^. In other moths as *P. xylostella* and *M. brassicae*, the presence of (Z) 3-hexenyl acetate increases the number of flights to and landings on the volatile source^25,26^. We suggest to test (Z) 3-hexenyl acetate alone either in a wind tunnel or in the field. It has been reported that recognition of the host plant by insects may be due to a single compound or to a mixture of compounds^1,28^.

In this study, *C. decolora* mated females are less oriented towards host plants that present signs of damage. Possibly isothiocyanates found in the headspace of damaged cabbage plants^27^ are induced by various factors like mechanical damaged, attack of pathogens, insects, and herbivores^29^. Isothiocyanates may affect the growth and development of the offspring by avoiding larvae competition for food and the risk of parasitism or predation^15,16^. In some moths, such as *T. ni* and *Ostrinia furnacalis* Guenée, damage by conspecific larvae to the host plant decreased attraction and oviposition^30,31^, in others moths as *Pieris brassicae* Linnaeus and *Mamestra brassicae* Linnaeus mated females were repelled by volatiles from cabbage plants damaged by conspecific larvae that attract parasitoids as *Trichogramma brassicae* Bezdenko and *Cotesia glomerata* Linnaeus^32,33^. Contrary to our findings, mated *M. brassicae* females were attracted to mechanical damaged plants releasing isothiocyanates^25^, and *Helicoverpa armigera* Hübner^34^ and *P. xylostella*^26^ were attracted to larvae damaged plants.

It is not clear if an undamaged plant really exists in nature; we used the term “undamaged” to name those plants whose leaves present no mechanical or herbivory damage. As plants release VOCs all year round, our study aims to demonstrate that generalist insect pests as *C. decolora* discriminates among those VOCs from damaged and undamaged host plants and this discrimination is very important when plants suffer from herbivory. A damaged plant is not optimal for feeding or oviposition, so laying eggs on a host or around it is a good strategy for survival increasing the biological fitness of the offspring^35^.

Studies in insect pests regarding the olfactory cues involved in the search of its host plant and the modification of this behavior due to the effect of damage to the plant, such as in this research, are important given that the knowledge generated should be considered for the monitoring and integrated management of *C. decolora* populations, where in addition to sexual pheromones that only capture males, host volatiles could be incorporated for attracting females.

## Methods

### Insects

Insects were obtained from a *C. decolora* colony at “Centro de Desarrollo de Productos Bióticos, IPN”, Yautepec, Morelos, Mexico; maintained at 22 ± 3 °C, 60 ± 3% RH and in a reverse 12 L:12D photoperiod. Larvae were fed a standard diet for Lepidoptera^36,37^ and adults a 50% sugar solution on a cotton pad. The adults were kept in acrylic transparent 20 × 20 × 20 cm boxes. Adults that emerged from pupae weighing 0.35 to 0.45 g were used in the experiments to remove any influence of weight on moth response to cabbage volatiles^38^.

A similar number (8–12) of 3 d old males and 3 d old females were placed together in the aforementioned box and observed until mating. After copulation, each female was individually kept in a 20 cm × 10 cm (height x diameter) transparent cylindrical container until needed. Mated 4–6 d old females were used for experimentation as a previous bioassay showed these had the best attraction-response to host plant volatiles^39^. After testing, to confirm that successful mating had taken place, the *bursa* *copulatrix* of each female was dissected and checked for the presence of a spermatophore. Data from females that had not mated successfully were discarded.

### Cabbage plants and headspace collection

Cabbage plants were grown in a greenhouse at 20 ± 2 °C, 60 ± 3% RH, and a 13 L: 11D photoperiod. The plants were placed in plastic pots (20 cm height and 25 cm diameter) containing sterile soil and were used when 30–40 d old, before flowering and with a fresh plant weight of approximately 120 g.

Cabbage volatiles were collected from the air that had been passed over an undamaged cabbage plant inside a glass chamber of 30 cm length and 20 cm diameter (modified from Geervliet *et al*.^40^), an acrylic plate placed in the airflow entrance held the plant by the stem, raising it above the soil. A glass pipette (13 cm length × 0.6 cm external diameter), containing 250 mg of Super Q (80 ∕ 100) adsorbent material (Alltech Assoc, Inc., Deerfield, Illinois, USA), was placed at the airflow exit. The glass chamber was connected to a vacuum pump (Welch® Vacuum Pumps and Systems, Gardner Denver Thomas, Inc., Houston, Texas, USA) that provided airflow regulated to 1,000 mL / min by a flowmeter (Cole Parmer, Ev-03217-06, Cole-Parmer Instrument Company, Vernon Hills, Illinois, USA). An activated carbon filter at the air pump intake was used to clean the air.

Volatiles were collected for 3 h (19:00 to 22:00 h corresponding to the flight time of the moth) on three consecutive days from 1 plant every day. Each volatile sample was eluted with 1 mL of hexane (HPLC, JT Baker, ®, Chemical Company, New Jersey, USA). Daily samples were pooled together and concentrated to 300 μL with a nitrogen stream and stored in a brown vial at −4 °C until use. A control sample was obtained using an empty chamber.

We tested three different headspace volatile compounds from: (a) undamaged cabbage plants, (b) larvae-damaged plants (10 third-instar larvae placed on the cabbage plant for 30 min; after that time, larvae and larval frass was removed from the plants and we waited 5 min before collecting volatiles) and (c) mechanical-damaged plants (stainless steel scissors, longitudinal cuts of 10 mm of 4–5 leaves of the plant done 5 min before volatile collection).

### Gas chromatography - Mass spectrometry (GC-MS)

A GC-MS (HP 6890/5972, Agilent, USA) was used to analyze and identify the chemical profile of the cabbage volatile headspace. A sample of 2 μL of each extract of the cabbage volatile headspace obtained was analyzed by GC-MS. The samples were analyzed with a non-polar HP 5 MS column (30 m, 250 μm internal diameter and 0.25 μm film thickness) (Agilent, Palo Alto, CA). The initial oven temperature was 60 °C and increased 4 °C/min to 220 °C^41^. We used helium as carrier gas at a constant flow rate of 1 mL/min while the temperature of the injector was 225 °C and the temperature of the auxiliary was 250 °C. The injector was used in the splitless mode for 20 s. The MS worked with electronic ionization (70 EV), in SCAN mode and a mass range of 35 to 550 AMU. The compounds were identified by retention times and their Kovats retention index, and the spectral comparison against the mass spectral library^42^ of the MS and synthetic standards (Sigma Aldrich ®, Toluca, Mexico).

The synthetic standards were: 6-methyl-5-hepten-2-one, (purity 99%), (Z) 3-hexenyl acetate (purity 98%), limonene (purity 98%), nonanal (purity 98%), Decanal (purity 98%), tetradecane (purity 99.5%), pentadecane (purity 98%), hexadecane (purity 99%) and heptadecane (purity 99%), all of them from Sigma Aldrich®, Toluca, Mexico.

### Wind tunnel bioassays

Moth response to olfactory stimuli was observed in a Plexiglas wind tunnel (180 length × 80 height × 80 width cm). An extractor (Frequency Inverter CFW-08 Software 4.1×, Minneapolis, USA) generated an airflow of 0.4 m/s cleaned by an activated carbon filter. An anemometer (Sper Scientific 840003, Taiwan) at 30 cm above the tunnel floor measured wind speed.

Moths were tested from 8:00 to 10:00 am, in their scotophase reversed with respect to the natural light cycle in order to allow bioassays during the day at 20 ± 3 °C, 60 ± 5% RH and under 3 red lights (20 watts, Philips® Mexico).

A 2 × 2 cm filter paper (Whatman # 1 ® 2 V, Merck KGaA, Darmstadt, Germany) with 10 μL (equivalent to 12 g of plant) of the stimulus to test (cabbage headspace from damaged plants [larval and mechanical], undamaged plants and hexane as a solvent control) was placed at the upwind end of the wind tunnel. After 20 s, a female was released downwind into the wind tunnel and observed for 300 s. After each test, clean air was pumped into the wind tunnel for 300 s.

Moth response to the stimulus was recorded as partial flight (oriented, but only through a part of the tunnel) and “complete flight” (if close to the source). Additionally, the number of females displaying their ovipositor was recorded. A total of 10 mated females were tested for each treatment. Females were used once and discarded.

A mixture of synthetic standards was prepared according to the relative concentration of each compound detected in the headspace from undamaged cabbage therefore, 100 µL of this mixture contained 6-methyl-5-hepten-2-one, (72.90 pg/μL), (Z) 3-hexenyl acetate (61.77 pg/µL), limonene (783.30 pg/µL), nonanal (744.45 pg/µL), decanal (197.23 pg/µL), tetradecane (87.27 pg/µL), pentadecane (83.92 pg/µL), hexadecane (90.57 pg/µL) and heptadecane (322.32 pg/µL). This mixture of synthetic standards proved to be as attractive to the mated females as the headspace volatiles from undamaged cabbage plants (n = 10 mated females) in the wind tunnel. For each moth, 10 μL and the same wind tunnel conditions mentioned above were used.

### Electroantennography (EAG)

EAG responses were conducted using Syntech EAG equipment (Kirchzarten, Germany). A recently dissected female’s antenna was mounted between 2 silver electrodes using conductor gel (Sigma gel, SYNTECH, Spectra 360, Parker, Orange, N.J. USA). The signal generated by the antenna was transmitted to an IDAC-2 amplifier and observed on a monitor with software (SYNTECH EAG PRO 2.0, 2005, SYNTECH, HILVERSUM, Netherlands) for EAG recording and analysis. A constant flow of humidified pure air (0.7 L/min) provided by a pump (stimulus controller SC-55) was directed onto the antenna through a glass tube (diameter 10 mm). To present a stimulus, a pipette tip containing the stimulus (2 μL of cabbage headspace was placed on filter paper 1 × 0.5 cm, Whatman No. 1) was inserted through a side hole located at the midpoint of the glass tube. The outlet of the glass tube was positioned approximately 2 cm from the antenna. Humidified pure air flowed at 0.5 L/min through the pipette during stimulation. The stimulus lasted 1 s. The solvent was allowed to evaporate for 20 s before testing and 120 s elapsed between stimuli. In these bioassays, the antenna depolarization response to hexane as control was subtracted from the response to each cabbage headspace before analysis. All stimuli were applied once per antenna using one antenna per moth.

Antennal depolarization responses of mated females were compared between the mixture of synthetic compounds, headspace undamaged cabbage plant, and hexane as control (n = 10 mated females).

### Data analysis

Female wind tunnel behavior was analyzed using a χ^2^ test with Yates correction, female EAG data were analyzed by ANOVA followed by Tukey’s test. Sigma Plot 11 (Systat Software, Inc., San Jose California USA) was used for all statistical analyses and the rejection probability was set at 0.05.
